# Exposure to linguistic labels during childhood modulates the neural architecture of race categorical perception

**DOI:** 10.1038/s41598-019-54394-6

**Published:** 2019-11-28

**Authors:** Susanna Timeo, Giovanni Mento, Erica Fronza, Teresa Farroni

**Affiliations:** 1 Department of Psychology and Cognitive Science, University of Trento, Trento, Italy; 2 Department of General Psychology, University of Padova, Padova, Italy; 3 Department of Developmental and Social Psychology, University of Padova, Padova, Italy

**Keywords:** Neural patterning, Perception

## Abstract

Perceptually categorizing a face to its racial belonging may have important consequences on interacting with people. However, race categorical perception (CP) has been scarcely investigated nor its developmental pathway. In this study, we tested the neurolinguistics rewiring hypothesis, stating that language acquisition modulates the brain processing of social perceptual categories. Accordingly, we investigated the electrophysiological correlates of race CP in a group of adults and children between 3 and 5 years of age. For both groups we found a greater modulation of the N400 connected with the processing of between category boundaries (i.e., faces belonging to different race groups) than within-category boundaries (i.e., different faces belonging to the same race group). This effect was the same in both adults and children, as shown by the comparable between-group amplitude of the differential wave (DW) elicited by the between-category faces. Remarkably, this effect was positively correlated with racial-labels acquisition, but not with age, in children. Finally, brain source analysis revealed the activation of a more modularized cortical network in adults than in children, with unique activation of the left superior temporal gyrus (STG) and the inferior frontal gyrus (IFG), which are areas connected to language processing. These are the first results accounting for an effect of language in rewiring brain connectedness when processing racial categories.

## Introduction

When we look at a face, we can almost immediately categorize its racial belonging^1^. This is because race is a fixed and salient characteristic of every person we meet. There is consistent evidence that race is perceived categorically rather dimensionally, so that faces belonging to a racial continuum are perceptually grouped into discrete categories^2–4^. This implies that, among a cross-racial continuum of faces, between-category differences are exaggerated and within-category differences are diminished, in order to create separated racial categories. As a socially relevant implication of this, race categorization may implicitly influence face perception leading to stereotypes and prejudices, thus biasing everyday interpersonal interactions.

While categorical perception (CP) seems to be well established in adults, we have a very coarse knowledge about its ontogenetic trajectories. As well, we do not know to what extent the developing cognitive system is sensitive to cultural factors, such as race label acquisition, in constructing the capacity to extract categorical meaning from face stimuli or whether this ability is independent from any cultural bias. Indeed, categories are usually defined by labels, which are often linguistic nouns. Hence, when a name is given to a group of stimuli, this creates a new category. In this view, it has been suggested that perception of social groups may be guided by top-down processes as language or cultural norms^5^. The efficacy of using linguistic to improve face recognition has been demonstrated also for 9-month-old infants^6^. Nonetheless, it has also been proposed that low-order visual phenomena as face color and shape might also bias face perception from very early developmental stages^7^. This account is supported by studies showing that infants as young as 3 month-old do show preliminary preference for^8^ and better recognition of own-race faces^9^. A recent study also showed that even 6-month-old infants, similarly to adults, perceptually create racial categories^10^. In other words, both low (perceptual) and high (cultural) order processes have been endorsed as possible routes constraining face categorization during development.

A recent theoretical model, the *neurolinguistic rewiring hypothesis*^11^, proposed a developmental account of CP in the attempt to reconcile these opposite findings, by holding that in preverbal infants and in children who do not possess a label for a specific category, CP may rely on perceptual cues. Instead, in adults and children who have learned the categorical labels, CP would be guided by brain areas subtending linguistic and high-order functions. In these populations, the categorical labels may shape different categories and thus modulate between-category distinctions. In effect, behavioural studies have highlighted the flexibility and mutability of race categorization and biases in adulthood^12^. In this perspective, the investigation of the neural mechanisms subtending CP in different developmental populations would provide more information than simply comparing their behavioral performances (as these may be similar). The hypothesis underlying the present study is that race categorization may be influenced by the acquisition of racial labels early across development, so that when a category label is learned, it may influence the categorization process. More specifically, the first hypothesis is to find evidence, at the electrophysiological level, of a CP effect for both children and adults. In this respect, we expect to find a category-dependent ERP modulation, with higher discrepancy for between- then for within-category comparisons. Furthermore, following a neurocostructive account, the second hypothesis is that the progressive emerging of CP as a function of racial label acquisition would be supported by a gradual cortical specialization leading from a distributed to a modularized network from childhood to adulthood^13–15^. Specifically, for adults we expect to find a greater activation of regions connected to language processing, mostly localized to left temporal lobes. Moreover, we expect to find that race-label acquisition in children would correlate with higher activation of linguistic-related areas during race CP.

**Figure 1:**
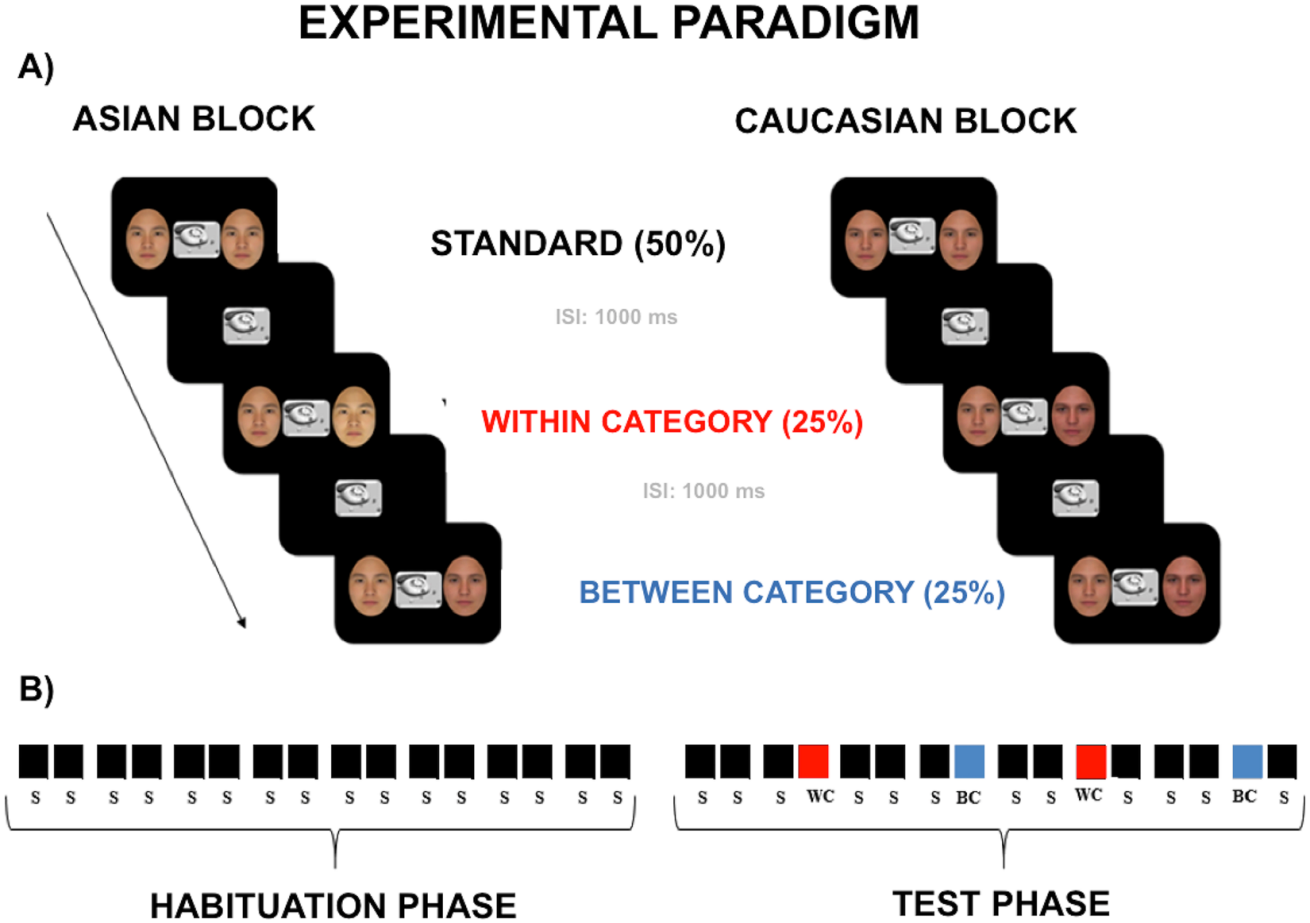
Multi-feature visual oddball. (**A**) Example of experimental trials for the Caucasian Block. (**B**) Example of quasi-random trial sequence.

To test these hypotheses, we compared the brain activation of a group of children (3 to 5 years) and adults during an implicit race CP task, illustrated in Fig. 1. Participants were presented with bilateral couples of faces morphed from a racial continuum (Caucasian-African) while they were watching a central movie. The race categorization was manipulated using a multi-feature oddball paradigm^16,17^, which included the presentation of identical faces (e.g., same race and features) in the 50% of the trials (standard condition). In the remaining trials participants were presented with faces that could belong to the same standard race category but with different physical features (within-category - WC - deviant; 25%) or to different race categories (between-category - BC - deviant; 25%). The morphing gap of both the WC and BC couples was identical (30%) in order to maintain the same perceptual distance. In this perspective, different responses for WC and BC would uniquely be ascribed to CP effects. During the task, the high spatial resolution EEG was recorded with the purpose of unveiling the spatio-temporal properties of the brain activity underpinning CP. Specifically, the N170 and the N400 event-related potential (ERP) components were targeted as two neural signatures reflecting structural and semantic face encoding, respectively. The difference wave (DW) was also obtained as an implicit measure of effective CP. This component has been purposely measured since it provides a relative measure of the category-dependent ERP modulation regardless the absolute amplitude magnitude of the scalp responses, that may show age-related morphological differences (deHaan, 2007).

## Results

**Figure 2:**
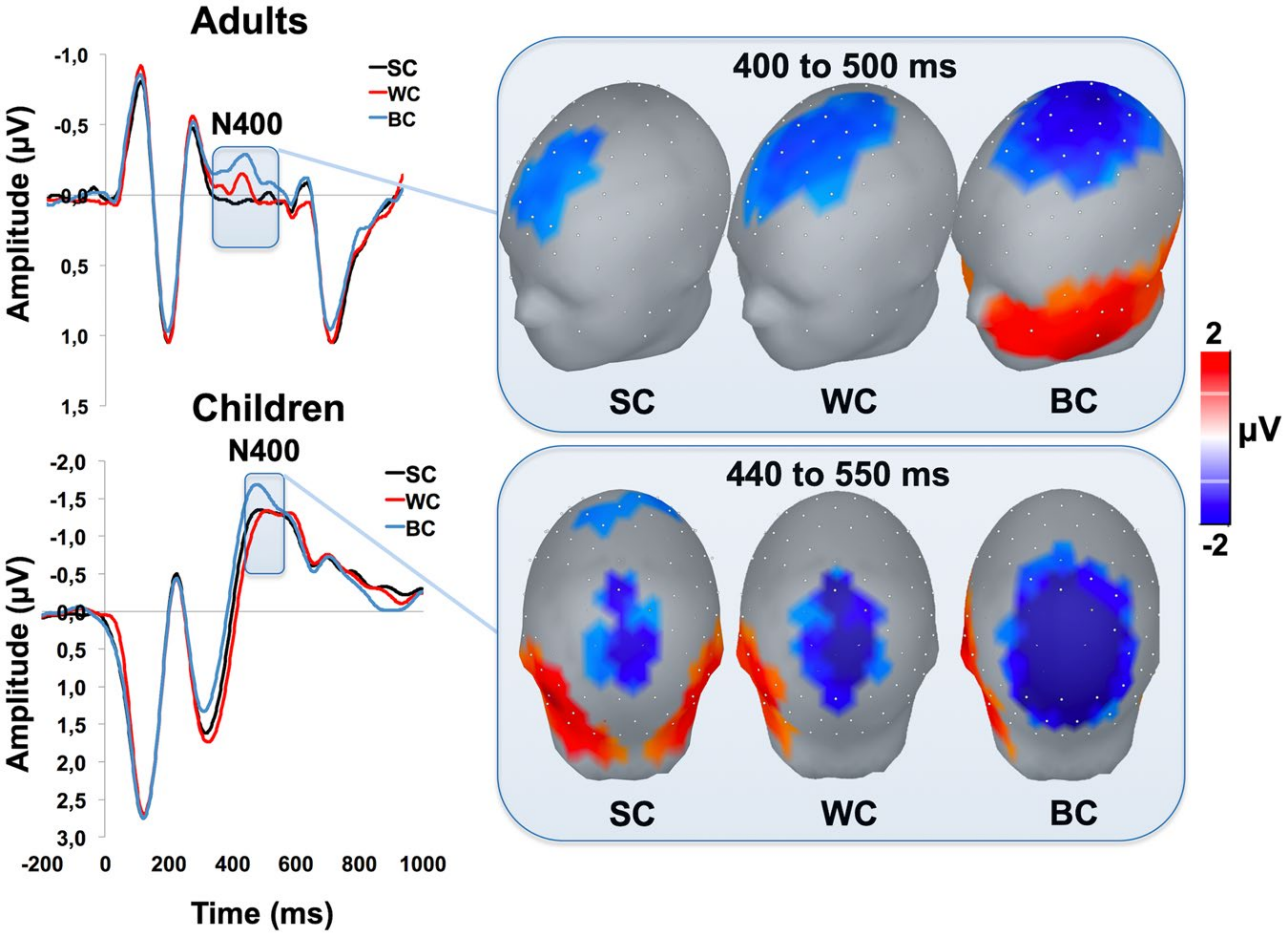
Brain responses induced by perceptual categorization. The picture shows the ERP elicited by faces belonging to standard (SC), within (WC) and between (BC) category in adults (top-left) and children (bottom-left). The grey bar on the x-axis represents the temporal extent of statistical significance for each cluster. The right part of the picture shows the three-dimensional scalp map reconstructions of the electrical voltage averaged across the time windows showing significant N400 effects in adults (top-right) and children (bottom-right). In both groups significant electrodes are grouped into distinct spatiotemporal clusters showing positive (in red) or negative (in blue) amplitude differences induced by perceptual categorization. The spatial distribution of the electrodes exceeding the critical t-score threshold for statistical significance is fronto-central in adults and posterior in children.

Neither adults nor children exhibited WC- or BC-related N170 effects. An additional analysis showed no significant N170 modulatory effects induced by the race itself when comparing the brain responses evoked by the Asian *vs*. the Caucasian standard faces. By contrast, as shown in Fig. 2, a larger N400 amplitude in response to BC as compared to either WC or SC faces was found in both groups (all t_s_ > ± 2.14; Family Wise Error corrected p < 0.05). No significant differences were found between SC and WC in both groups neither for the N400 nor for the DW.

Furthermore, the N400 peaked earlier in adults as compared to children for the SC (t(37) = −3.46; p = 0.001; Cohen’s d = −1.12), the WC (t(37) = −3.27; p = 0.002; Cohen’s d = −1.06) and the BC (t(37) = −2.64; p = 0.01; Cohen’s d = −0.85) conditions.

**Figure 3:**
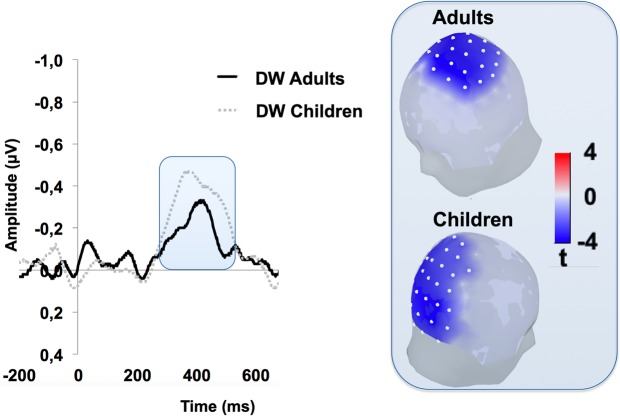
The left panel shows the modulation of ERP activity induced by categorical perception. The waveforms represent the difference Wave (DW) calculated by subtracting the ERP activity elicited by standard faces (SC) from the one elicited by faces featuring other-race connotations (BC) for adults (black continuous line) and children (gray dashed line). The right panel displays the scalp localization of the DW in the two groups.

Noteworthy, despite the overall between-group differences in face-related ERP responses in terms of latency (the N400 was slightly later and larger in children than adults) due to maturational issues, the extent to which it was affected by categorical perception was quite comparable between children and adults. Indeed, both groups exhibited a similar DW obtained as the differential ERP activity between BC and SC condition. Yet, the DW showed age-related differences in scalp distribution, spreading over fronto-central and occipito-parietal electrodes in adults and children, respectively (Fig. 3).

**Figure 4:**
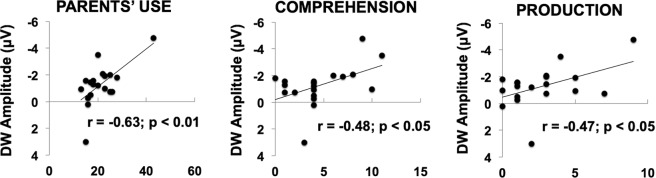
Correlations between the DW with parent’s assessment of children race-related language. The picture shows the correlation between the children’s DW for faces featuring other-race connotations (BC) and the parent’s use of racial labels (left panel), the child’s comprehension (central panel) and the child’s production of racial labels (right panel), as reported by parents.

As a remarkable finding, the perceptual categorization in children was predicted by the parents’ rating on their linguistic competence about race. Namely, a significant correlation was found between the magnitude of the DW for BC stimuli and the score obtained in the questionnaires relatively to the parents’ frequency use of racial labels (r = −0.63; p < 0.01), the child’s comprehension (r = −0.48; p < 0.05) and production (r = −0.47; p < 0.05) of racial labels (Fig. 4). No significant correlation was found between DW amplitude and age (r = −0.10; p > 0.05), nor between DW and the children’ naming and comprehension tests (r = −0.12; p > 0.05).

**Figure 5:**
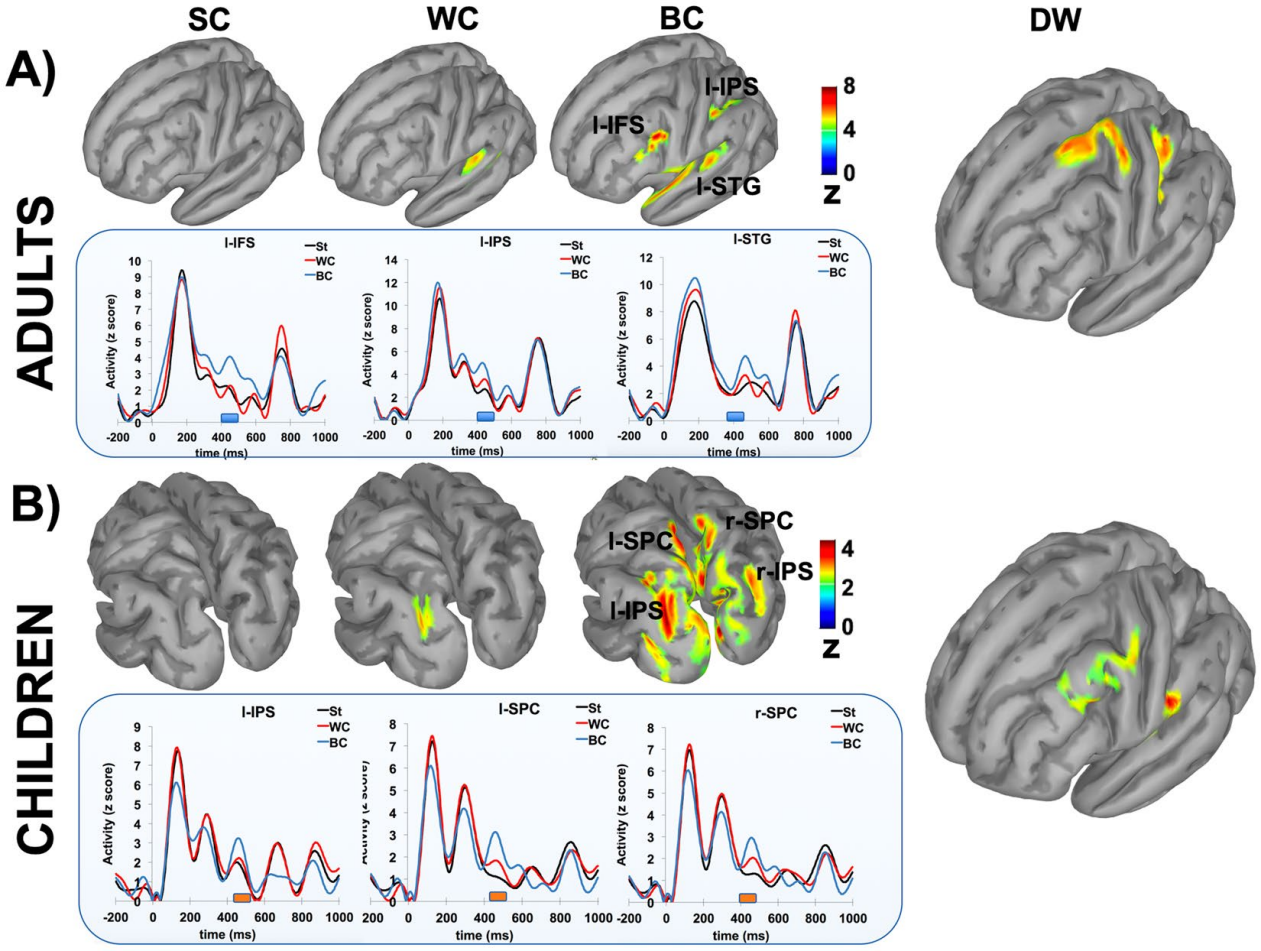
The CP cortical networks. The upper part of each panel shows the reconstruction of the cortical maps for each category and relatively to the temporal window showing significant scalp-related N400 amplitude effects for both adults (**A**) and children (**B**). For both groups, the lower part of each panel shows the temporal dynamics of the activations of the relevant cortical ROIs in relation to standard, within and between stimulus categories. Colored bars over the x-axis indicate the significant temporal windows for N400 components for both adults (blue bars) and children (orange bars). Cortical activations were adjusted using a threshold of 50% of the maximum amplitude and a size of at least 20 vertices.

The brain source reconstruction of the ERP significant effects showed that different cortical activations underpinned the scalp-related differences observed between groups. Specifically, the adults exhibited a circumscribed activation neatly localized within left temporal lobe (including the inferior/superior temporal gyri). By contrast, the children displayed a more distributed posterior network encompassing occipito-parietal areas only partially left-lateralized (Fig. 5).

## Discussion

The present study has investigated the neurocognitive development of the CP of social stimuli, namely ethnic groups, and the impact of racial labels acquisition on it. Taken together, our findings suggest that CP of race is early established during childhood and operates in the absence of overt attention to faces, since we were able to observe N400 modulation in response to laterally displayed, cross-race faces while participants’ attention was focused on a central cartoon video. As expected, CP took place after structural encoding of face features and was reflected by a specific neural marker (i.e., N400) in both children and adults. Indeed, our results have shown neither BC nor WC modulatory effects on the N170, an ERP component reflecting the early, structural encoding of facial features^18^. Instead, the N400 has been traditionally linked to domain-general semantic categorization, since it is elicited by both linguistic (i.e., incongruent words in a sentence) and non-linguistic stimuli as faces^19^ or bodies^20^. Some authors have also hypothesized that the N400 derives from a multimodal neural network, which synchronizes stimuli coming from different modalities to create an integrated conceptual representation^21^. In this perspective, race CP seems not to be directly supported by lower perceptual processes (the N170 is not affected by race CP), but instead by the later integration of different perceptual and cognitive inputs (including semantic linguistic categories). Moreover, this process seems to be, if not automatic, at least implicit. In fact, the presentation of stimuli was outside the foveal visual area of participants, so these were computed without the need of overt attention. Noteworthy, neural markers of race CP have been found for both adults and children, leading to support the early development of this phenomenon. This finding is consistent with empirical evidence that (1) infants as young as 3 months showed to distinguish faces according to their racial features^9,10^ and (2) 6-month-old infants were able to perceptually categorize faces by race^10^. However, the extraction of racial categories outside a continuum (race CP) has never been tested before in a developmental population. Our results suggest that, between 3 and 5 years of age, children have developed an implicit CP of racial categories. Most importantly, the elicitation of the N400 suggests this representation not to be solely perceptual in nature, but also to involve semantic processes.

Remarkably, the most intriguing finding regards the relation between race CP and race-label knowledge in children. In this population, the absolute magnitude of the between-category DW, the neural index of CP, was correlated to the level of race-related language acquisition as rated by parents. Although this finding deserves further empirical evidence, it is in line with the neurolingusitc rewiring hypothesis^11^, which states that the acquisition of category labels does not change the process of CP by itself, but instead, this is strengthened through the recruitment of high-order neurocognitive mechanisms. In effect, we found a correlation between children’s’ linguistic knowledge and the differential waveform (DW) of the N400 component, which, in turn, is related to the semantic construction of categories. In this sense, the greater race-related linguistic knowledge seems to support a stronger semantic construction of the racial categorical boundary. Overall, the acquisition of language might recruit new or additional neural pathways in order to accomplish the same behavioural CP process. However, CP seems to go through a further cortical specialization across development implying the shaping of areas exhibiting domain-specific activity. Whereas children seem to engage more occipito-parietal areas, closely linked to perceptual processes^22^ (i.e., visual attention), only adults seem to localize their CP-related activation in the left superior temporal gyrus (STG) and the inferior frontal gyrus (IFG). All these brain areas have been for long connected to speech perception and production^23–26^. In this perspective, these findings seem to point out to a progressive neuropsychological specialization of CP, from a more distributed and perception-based to a more focused and linguistic-driven process. As stated by the neurolinguistics rewiring hypothesis, if the acquisition of labels leads linguistic areas to take control over perceptual ones, then changing labels would end up in changing perception. In this perspective, future studies should test whereas, after having learned racial labels, the perpetual modulation of racial categories in children becomes more fluid and malleable.

As a limitation, this study only examined differences between groups (i.e., children vs adults and children who do or do not know race labels) instead of longitudinally comparing the performance of children before and after the acquisition of category labels. In fact, this work has demonstrated that the exposure to race labels and their comprehension or production was related to children’s concurrent processing of race categories, but it did not examine whether these variables related to intra-individual change or learning about race categories. We hope future studies will disentangle these aspects of label acquisition.

Overall these results lead to new theoretical questions. First of all, are these findings specific for the ethnic or social categorization or are they general for all type of visual stimuli? In effect, racial categories are based on perceptual differences, but belong to the domain of social stimuli, which are usually perceived differently than other physical objects^27^. However, previous behavioural studies on color CP have highlighted a similar pattern of specialization, driven by colour-label acquisition^28^. In this perspective, it remains still unclear whether CP of physical objects would activate the same or different brain areas compared to CP of social stimuli.

A second issue regards the evaluation of the impact of linguistic labels on CP. Would nouns only act to reinforce a pre-existing perceptually-driven category or might they even change the CP process? In other words, if children were taught names stressing different cross-category boundaries (i.e., the size of eyes), would they show different CP effects? This question lays on the long-lasting debate about the influence of culture (specifically language) on human’s perception (i.e., Sapir-Whorf hypothesis). In the domain of colour perception, for example, researchers have shown that tribes who do not possess names for some colour categories (i.e., only one label for blue and green) did not show CP for those categories^29^. For race categorization, one study demonstrated that changing categorical grouping (from race to the belonging to different University Institutes) also changed subsequent mnemonic performance on the face^12^. Would it be possible to make race-blind people by changing their linguistic construction of groups? This and other questions should be tested by future research.

Overall, this research has investigated the effect of the acquisition of language in creating race-related perceptual categories and how this modulates the underlying neural architecture across development. Our findings support the hypothesis that linguistic labels impacts the formation of categories, by recruiting high-order, language related brain areas, although further studies with a larger sample size are needed to corroborate this hypothesis. Taken as a whole, these findings support a neurocostructive account of race-related CP.

## Methods

### Participants

A total of 26 Caucasian children aged between three and five years took part to the study. Two participants were Caucasian-African mixed-race. We controlled the analyses for these two participants, but the results did not change so we left them into the sample. The exclusion criteria for study recruitment included the presence of preterm birth, clinical complications developed before or after birth, together with the presence of severe neurological or psychiatric familiar disorders. From the children group, eight participants were excluded because of insufficient number of trials caused by excessive EEG movement artefacts or fussiness. The remaining 18 children were between 3 and 5 years of age (mean age = 50,95 months; SD = 9,32; range: 36–71; F = 12). The same experiment was administered to 26 healthy, Caucasian adults, recruited among students of the University of Padua, with normal or corrected-to-normal vision and no neurological and psychiatric disorders. Six were excluded because a sufficient number of trials could not be extracted due to excessive EEG movement artefacts. The remaining 20 participants (mean age = 22.6; SD = 3,23; range = 19–29; F = 11) were included in the study as the adult control group. The study was approved by the ethical committee of the School of Psychology of the University of Padua (prot. 1826). All adult participants and children parents gave informed written consent for the participation at the study. All methods were performed in accordance with the relevant guidelines and regulations.

### Stimuli

Pictures displaying couples of 16,5 × 12,5 cm (visual angle: 15°) color Asian and Caucasian mixed-morphed faces (Winmoprh 3.01, DebugMode) were used as experimental stimuli. Specifically, four Asian face identities from the oriental face database collected under the research of the Artificial Intelligence and Robotics (AI&R) of Xi’an Jiaotong University and four Caucasian face identities from the Minear and Park face database^30^ were averaged and used to generate a protoype face for each race (one Caucasian and one Asian face). Then, a symmetrical continuum of 21 images (morphs) that represented gradual transitions from the prototype Asian to the prototype Caucasian face in steps of 5% (from 0% to 100%) was generated.

In addition, in order to attract participants’ attention to the centre of the screen, an animated cartoon, displaying a spaceship building objects, was played for all the experimental time. The cartoon, whose size was 14,5 × 7 cm (visual angle of 14°), was chosen on the basis of its attractiveness for children and was also grey-scaled with Windows Movie Maker 2016. No facial features were present in the animated cartoon in order to avoid any effect of the video on face-related ERP components. Visual stimuli were displayed on a 23-inch Benq LCD monitor with a resolution of 1920 × 1080 (16:9 of aspect ratio) and a refresh rate of 60 Hz. Their presentation was controlled by the software E-Prime 2.0 (Psychological Tools).

### Experimental paradigm

The stimuli were delivered by using a multi-feature oddball paradigm^16,17^ (Fig. 1a). The advantage of using this paradigm compared to the classic Oddball paradigm^31,32^ is to allow the investigation of implicit, perceptual categorization of sensory stimuli while ruling out the confounding effect of the unbalanced trial probabilistic distribution between the standard and the deviant category, which in turns may result in a biased ERP signal-to-noise ratio.

Two different types of blocks were administered, defined as the Caucasian and the Asian block. In the Caucasian block-type both the St and the WC stimuli were Caucasian faces, whereas the BC stimuli were Asian faces. Vice-versa, in the Asian block-type both the St and the WC stimuli were Asian faces, while the BC stimuli were Caucasian faces. Each pair of faces lasted on the screen 500 ms, followed by a 1000 ms inter-trial interval. The number of trials was balanced between the two blocks (for more details, see Supplementary Materials).

At the beginning of each block a habituation phase was delivered. This consisted of the presentation of 8 standard couples of standard faces showing same race features (SC), which may be either Caucasian or Asian, depending on the block-type. The habituation phase was immediately followed by the test phase, which consisted in the presentation of trials with same temporal structure as those in the habituation phase, but now including both standards and deviants faces (Fig. 1b). During the repetitive, lateralized delivering of the faces, a black and white cartoon was centrally and continuously played to capture the participants’ overt attention. Adult participants were warned to pay particular attention to the cartoon, because at the end they were asked to answer some questions about particular scenes of it.

At the end of the experimental task, we collected data on participants’ contact with other-race people and parents were asked to complete a questionnaire on their child’s knowledge of race-related language (see Supplementary Material).

### EEG recording and analysis

Continuous EEG data were collected and amplified using a Geodesic EEG system (EGI GES-300), through a pre-cabled high density 128-channel Hydrocel Geodesic Sensor net (HCGSN-128) with the vertex as reference point. A sampling rate of 500 Hz was used and the impedance was kept below 60 KΩ for each sensor. All data were lately processed offline using the toolbox EEGLAB applying a band-pass filter between 0.1–30 Hz. In line with our previous ERP studies on developmental population^33–35^, all participants were video-taped during the whole experimental session to monitor their gaze and manually reject every trial to which no attention was paid to. Epochs ranging from −200 to 1000 ms according to the onset of the faces were extracted on purpose of investigating the modulation of the ERP activity in response to racial features in the standard and deviant conditions. Electrodes presenting artefactual activity were eliminated and their activity reconstructed through spherical interpolation. Further manual inspection was done for each subject in order to eliminate those epochs that presented huge artefacts amenable to body movements. After interpolating bad channels and segmenting the data according to stimulus type, the Independent Component Analysis (ICA) was used to isolate and eliminate artefacts as eye movements and blinking. Epochs with potentials exceeding ±100 and ±200 µV at any electrode, in adults and children respectively, were further rejected. Then, the signal was average and baseline-corrected using the signal preceding 200 ms the onset of the stimuli. For the Standard Condition, a mean of 305 ± 8 and of 180 ± 23 artefact-free epochs were accepted for adults and children, respectively, whereas for the Deviant conditions, a mean of 151 ± 12 and of 90 ± 12 artefact-free epochs were accepted for adults and children, respectively. No significant differences were found in the number of trials accepted between the two deviant conditions in neither group (all p_s_ > 0.05). The EEG segments for standard and deviant conditions were averaged individually and ERPs components were grand-averaged for each subject and condition. The cortical maps of the temporal windows showing significant amplitude modulations due to experimental manipulations were reconstructed by using the Brainstorm software. For further details on ERP statistical analyses and brain source reconstruction see Supplementary Material.

## Supplementary information

Supplementary Info

## Data Availability

The datasets generated during and/or analysed during the current study are available from the corresponding author on reasonable request.
