# Supplementary material for: Exposure to linguistic labels during childhood modulates the neural architecture of race categorical perception
Source: Sci Rep. 2019 Nov 28;9:17743. doi: 10.1038/s41598-019-54394-6 (PMC6882795; doi:10.1038/s41598-019-54394-6)
Supplement: Supplementary file 1 — Supplementary Info [file 41598_2019_54394_MOESM1_ESM.docx]

**Supplementary material**

**Exposure to linguistic labels during childhood modulates the neural architecture of race categorical perception.**

Susanna Timeo^1,*^, Giovanni Mento^2,3,*^, Erica Fronza^3^ & Teresa Farroni^3^

^1^ Department of Psychology and Cognitive Science, University of Trento, Italy.

^2^ Department of General Psychology, University of Padova, Italy.

^3^ Department of Developmental and Social Psychology, University of Padova, Italy.

^*^ These authors contributed equally to the work.

**Stimuli creation**

The stimuli were selected from the oriental face database collected under the research of the Artificial Intelligence and Robotics (AI&R) lab of the Xi'an Jiaotong University and four Caucasian face identities from the Minear and Park face database^1^. The Minear and Park Face Database was created at the University of Michigan and is freely available for research purposes (<http://agingmind.utdallas.edu/download-stimuli/face-database/>). As stated by the authors, all participants signed a release allowing their faces to be used in scientific publications.

Four faces with the same morphing distance (30%) were selected from the continuum (95% Asian-5% Caucasian, 65% Asian-35% Caucasian, 35% Asian-65% Caucasian and 5% Asian-95% Caucasian, respectively). The combination of the adjacent faces created one between- (BC) and two within-category (WC) couples (one for the Asian and one for the Caucasian category). Moreover, a standard (ST) couple was created with both faces (left and right) displaying the same identity and race. Specifically, ST faces were either 65% Asian-35% Caucasian (ST for Asian block) or 35% Asian-65% Caucasian (ST for Caucasian block) (see fig. 1S for an example of Caucasian Standard faces). This percentages of morphing were chosen because in this way the ST pairings would have been equidistant from the between and within categories.


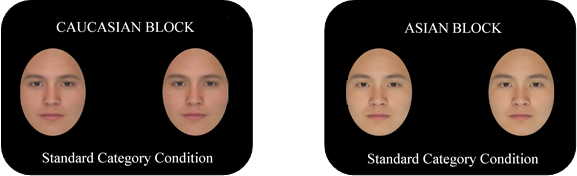


**Figure 1S.** Example of the Standard Conditions

In the WC condition one of the two standard faces (either the left or the right; 50% counterbalanced) was substituted by a face of the same race of the standard (i.e., Caucasian face in the Caucasian block and Asiatic face in the Asiatic block) but with a different percentage of morphing (95%; see Fig. 2S for an example of Caucasian WC deviant). This was done to induce a categorization to the same racial group as the ST one.


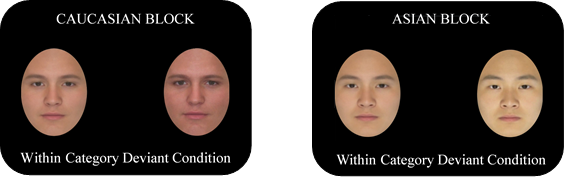


**Figure 2S**. Example of the Within Category Deviant conditions.

In the BC condition one of the two standard faces (either the left or the right; 50% counterbalanced) was substituted by an other-race face with an identical percentage of morphing of the standard one, namely 65%, but with the opposite contribution of races (see fig. 3S for an example of Caucasian BC deviant). Because the new face crosses the 50% morphing race-boundary, as compared to the standard, it should elicit the implicit perception of a different ethnical category.


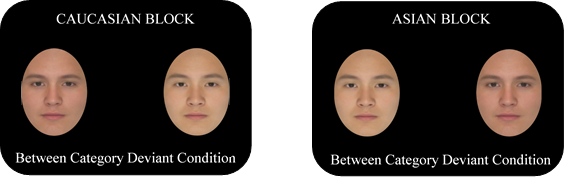


**Figure 3S**. Example of the Between Category Deviant Condition

**Oddball paradigm procedure**

In each experimental block, the St faces were presented in the 50% of the trials (standard condition). In the two deviant conditions (25% each) the faces could show either WC (12.5% left and right visual field changes, respectively) or BC (12.5% left and right visual field changes, respectively) feature changes. Namely, 52 face pairs were presented for the SC, while the WC and BC included 26 face pairs each. These three conditions were delivered in a quasi-randomized way so that each deviant stimulus did not appear two times in a row. The adult group was passively presented with a total of 6 blocks, equally divided between Asian- and Caucasian-type. The total number of trials was 624. The same was done for the children’ group in exception of the number of blocks that was reduced to four (i.e., two Caucasian and two Asian), for a total of 416 trials. The block order was counterbalanced across participants.

**Comprehension and naming assessment.** We created a brief questionnaire of race-related language assessment similar to other common communicative development inventories^1^. Parents were asked to rate how often they use 11 common racial labels ( i.e., Chinese, White, African, etc..) with their children on a Likert scale from 1 (never) to 5 (often). They were also asked to rate, for each of the 11 labels, whether the child comprehends and/or produces it. Ratings were summed up in order to create indexes of parental use, child comprehension and child’s production of racial labels.

In parallel, children’s comprehension and naming abilities were tested for racial labels using a procedure similar to Franklin and colleagues^2^. The cover story was that some people have got lost and the child should help the experimenter to find out were they came from. To test comprehension, three race-prototypical faces (Caucasian, Asian, African) were simultaneously presented to the child, who were asked to point out to the face corresponding to each race (Caucasian was substituted by Italian label). In order to test naming, each face was presented sequentially and the experimenter asked to the child “If this person is Italian/Asian/African) this one is…?”.

**Other-race contact**

Adults’ familiarity with Asian people was assessed with a scale adapted from Brown, Vivian and Hewstone^3^. First, participants were asked if they have ever had contact with people of “Asian descent”. Whereas they responded positively they were asked how many people of that descent they knew, from 1 to 7 = 7 or more people (familiarity). Subsequently, they were asked to think of the person of this descent they knew best and to rate how often they interacted with this person, from 1 = never to 7 = every day (frequency). They were then asked about the type of contact. On an eight-point scale they had to judge how much the relationship with this person was from 1 (a casual acquaintance, unfriendly, formal and competitive) to 8 (a close friendship, friendly, informal and cooperative). Finally on an eight-point scale from 1 (never) to 8 (always) they were asked how often they made references to one another's ethnicity in their encounters with this person and how much this person could be considered `typical' of his/her ethnic group (race typicality).

To assess children’s contact with other races, parents were asked whether their child knew kids of other ethnic groups and, if so, which ones (African, Asian, others). The familiarity with other races was calculated by summing up all known race, from 0 =no contact to 3 = maximum contact. Finally, parents were asked whether the child have started to note ethnic differences (i.e., if their child has ever asked why people are different).

**EEG-analyses**

**Statistical analyses**

To test for the presence of significant temporal and spatial ERP differences elicited by categorical perception a non-parametric, cluster-based permutation tests based on the cluster mass statistic using the original data and 1000 random within- participant permutations of the data were performed pair-wise among the experimental conditions^4^. Electrodes within approximately 1.5 cm of one another were considered spatial neighbours and adjacent time points were considered temporal neighbours. Pair-wise comparisons were performed. All pairs whose t-values were larger than a pre-determined threshold of ±2.14 (corresponding to a Family Wise Error corrected alpha value of .05) were considered significant. Moreover, the ERPs elicited by the presentation of the face couples were correlated with the scores obtained in the questionnaires. The Shapiro-Wilk test confirmed that all data were normally distributed (all Ws > 0.85; all ps >0.05). This allowed us to use a Pearson’s correlation. Both the peak amplitude and latency maxima of the ERP components of interest were extracted from specific time windows and electrodes according to both visual inspection of the grand average and literature (de Haan, 2007). The N170 analyses was conducted by measuring peak latencies and amplitudes within two specific time-windows: 130-210 ms and 190-300 ms for adults and children respectively. The electrodes number 58 and 96 of the Hydro-cell Geodesic Sensor Net montage were considered, corresponding to the T5 and T6 positions in the 10-20 system (Jasper, 1958).

The N400 was measured between 400 and 500 ms for adults and between 450 and 550 ms for children. According to the visual inspection of the grand average (Fig. 3) the peak latency and amplitude of this component were measured at the vertex electrode for adults and at the electrode 62 for children, corresponding to Cz and Pz respectively. The difference wave (DW) was extracted by subtracting the standard response from each deviant stimulus and measured in the same time windows and electrodes as the N400.

**Brain source reconstruction**

Although electrophysiological techniques are limited in their spatial resolution, the use of a high-density system allows to partially overcome this problem. In fact, thanks to the high-density electrode array (i.e., ≥ 128), it is possible to investigate the temporal dynamics of the reconstructed cortical activity by using a brain source analysis approach. The possibility to determine the brain sources underlying a specific electrophysiological component is useful to better understand its functional significance. The cortical generators underlying the ERP components of interest were reconstructed using the Brainstorm software package^5^ and adopting the same procedure as in our previous studies^6-8^. The conductive head volume was modelled according to the 3-spheres BERG method^9^. The solution space was constrained to the cerebral cortex, which was modelled as a three- dimensional grid of 15,028 fixed dipoles oriented normally to the cortical surface. The inverse transformation was applied to the Montreal Neurological Institute (MNI) canonical mesh of the cortex to approximate real anatomy. The inverse modelling was based on the low resolution tomography (sLORETA)^10^ solution implemented as a routine of the Brainstorm platform. For each participant the sources were projected to a standard anatomical template (MNI) and their activity was transformed in absolute Z scores relative to the baseline. The cortical activations relative to the time window showing significant ERP modulations were located according to the anatomical Desikan–Killany atlas^11^ adapted for cortical space solution.

1. Minear, M & Park D.C. A lifespan database of adult facial stimuli. *Behav Res Methods Instrum Comput.* **36**(4). 630–3 (2004).
2. Franklin A, et al. (2008) Lateralization of categorical perception of color changes with color term acquisition. *Proc Natl Acad* Sci USA:pnas-0809952105.
3. Brown R, James V, Hewstone M, Changing attitudes through intergroup contact: the effects of group membership salience. *Eur. J. Soc. Psychol*. **29**, 741–764 (1999)

# Groppe, D. M., Urbach, T. P. & Kutas, M. Mass univariate analysis of event-related brain potentials/fields I: A critical tutorial review. *Psychophysiology* 48, 1711–1725 (2011).

1. Tadel F, Baillet S, Mosher JC, Pantazis D, Leahy R.M. Brainstorm: a user-friendly application for MEG/EEG analysis. *Comput Intell Neurosci* **8,** (2011).
2. Mento, G. (2017). The role of the P3 and CNV components in voluntary and automatic temporal orienting: a high spatial resolution ERP study. *Neuropsychologia*, **107**, 31-40.
3. Mento G. & Valenza E. (2016). Spatiotemporal neurodynamics of automatic temporal expectancy in 9-month old infants. *Sci Rep*, **6**, 36525
4. Mento G. and Vallesi A. (2016). Spatiotemporally dissociable neural signatures for generating and updating expectation over time in children: a High Density-ERP study, *Dev Cog Neurosci*, **19**, 98-106.
5. Berg P. & Scherg M. A fast method for forward computation of multiple-shell spherical head models. Electroencephalogr. *Clin. Neurophysiol*. 90, 58–64 (1994).
6. Pascual-Marqui R. D. Standardized low-resolution brain electromagnetic tomography (sLORETA): technical details. *Methods Find Exp Clin Pharmacol*, **24**(Suppl D), 5-12. (2002).
7. Desikan R.S., Ségonne F., Fischl B., Quinn B.T., Dickerson B.C., Blacker D., Buckner R.L., Dale A.M., Maguire R.P., Hyman B.T., Albert M.S., Killiany R.J. An automated labeling system for subdividing the human cerebral cortex on MRI scans into gyral based regions of interest. *Neuroimage* **31**, 968–980 (2006).
